# Dietary Patterns and Cerebral Glucose Metabolism in Older Adults: Findings from the Western Australia Memory Study

**DOI:** 10.1002/alz70860_100615

**Published:** 2025-12-23

**Authors:** Carolina B Castro, Samantha L Gardener, Farzana Jahan, Juliana Chen, Belinda M. Brown, Ruey Loo, Kevin Taddei, Stephanie R Rainey‐Smith, Michael Weinborn, Ana Caroline R. dos Reis, Ralph N Martins, Hamid R Sohrabi

**Affiliations:** ^1^ Murdoch University, Murdoch, Western Australia, Australia; ^2^ Alzheimer's Research Australia, Perth, Western Australia, Australia; ^3^ Edith Cowan University, Perth, Western Australia, Australia; ^4^ Murdoch University, Perth, Western Australia, Australia; ^5^ The University of Sydney, Sydney, NSW, Australia; ^6^ Macquarie University, Sydney, NSW, Australia; ^7^ Murdoch University, PERTH, Western Australia, Australia; ^8^ Alzheimer's Research Australia, Perth, Western Australia, Australia; ^9^ Edith Cowan University, Joondalup, Australia; ^10^ The University of Western Australia, Perth, Western Australia, Australia; ^11^ State of Bahia University, Salvador, Bahia, Brazil; ^12^ School of Medical and Health Sciences, Edith Cowan University, Perth, Western Australia, Australia; ^13^ School of Psychology, Murdoch University, Murdoch, Western Australia, Australia

## Abstract

**Background:**

Alzheimer's disease (AD), the most common form of dementia, is marked by significant reductions in glucose metabolism. Such hypometabolism reflects underlying synaptic dysfunction, correlating with cognitive decline. Our study aimed to explore the impact of dietary patterns—specifically, the Western Diet and Prudent Diet—on change in glucose metabolism in brain regions associated with AD risk, [^18^F]Fluorodeoxyglucose positron emission tomography (FDG‐PET) imaging as a biomarker.

**Method:**

Longitudinal data from 133 cognitively unimpaired older adults were analysed from the Western Australian Memory Study. Participants underwent dietary assessment using the Cancer Council of Victoria Food Frequency Questionnaire and completed FDG‐PET imaging up to three times over 43 months. Dietary patterns were identified through principal component analysis, yielding two patterns—named Western Diet and Prudent Diet. Pattern scores were computed by summing food group intakes weighted by their respective factor loadings. Linear mixed‐effect models evaluated the association between dietary adherence and brain glucose metabolism, including potential confounders. The cohort was stratified by apolipoprotein E (*APOE)* ε4 carrier status, a genetic risk factor for AD, to investigate potential differing effects.

**Result:**

Adherence to a Western Diet, characterised by high sugars and saturated fats, was associated with a faster decline in glucose metabolism in the right fusiform gyrus among *APOE* ε4 carriers (β = ‐0.00012; SE = 0.00004; false discover rate adjusted *p* = .032), with no significant associations in *APOE* ε4 non‐carriers. Similarly, no significant associations were observed between the Prudent Diet, characterised by high intake of fruits, vegetables, and whole grains, and glucose metabolism, in both *APOE* ε4 carriers and non‐carriers.

**Conclusion:**

Our study highlights the potential detrimental impact of a Western Diet on brain glucose metabolism, particularly for individuals at genetic risk for AD. The decline in glucose metabolism in the fusiform gyrus, a region essential for cognitive functions like facial recognition, emphasises the role of diet in brain health. Future research should examine the mechanisms linking diet to neurodegeneration and explore dietary interventions as preventive strategies against cognitive decline and dementia.

